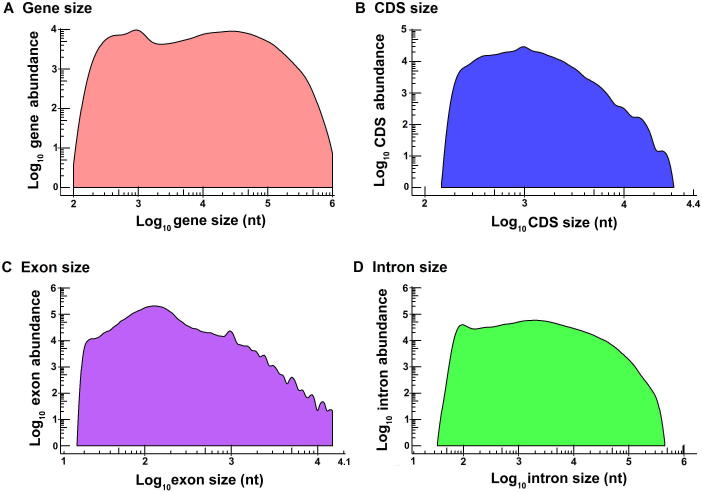# gFACs: Gene Filtering, Analysis, and Conversion to Unify Genome Annotations Across Alignment and Gene Prediction Frameworks

**DOI:** 10.1016/j.gpb.2019.04.002

**Published:** 2019-08-19

**Authors:** Madison Caballero, Jill Wegrzyn

**Affiliations:** Department of Ecology and Evolutionary Biology, University of Connecticut, Storrs, CT 06269, USA

**Keywords:** Genome annotation, Bioinformatics, Protein annotation, Gene prediction, Alignment

## Abstract

Published genomes frequently contain erroneous gene models that represent issues associated with identification of open reading frames, start sites, splice sites, and related structural features. The source of these inconsistencies is often traced back to integration across text file formats designed to describe long read **alignments** and predicted gene structures. In addition, the majority of **gene prediction** frameworks do not provide robust downstream filtering to remove problematic gene annotations, nor do they represent these annotations in a format consistent with current file standards. These frameworks also lack consideration for functional attributes, such as the presence or absence of protein domains that can be used for gene model validation. To provide oversight to the increasing number of published **genome annotations**, we present a software package, the Gene Filtering, Analysis, and Conversion (gFACs), to filter, analyze, and convert predicted gene models and alignments. The software operates across a wide range of alignment, analysis, and gene prediction files with a flexible framework for defining gene models with reliable structural and functional attributes. gFACs supports common downstream applications, including genome browsers, and generates extensive details on the filtering process, including distributions that can be visualized to further assess the proposed gene space. gFACs is freely available and implemented in Perl with support from BioPerl libraries at https://gitlab.com/PlantGenomicsLab/gFACs.

## Introduction

In the era of high-throughput sequencing, the size and complexity of the genomes assembled in recent years, have dramatically increased. Despite this, only a handful of the nearly 7800 eukaryote genomes in GenBank are resolved at, or close to, chromosome level [1]. In addition, over 85% of these genomes contain some type of gene annotation errors [2], [3], [4]. These challenges are likely to persist with projects, such as the Earth BioGenome Project, planning to sequence 1.5 millions of eukaryotic genomes in coming years [5]. Initiatives such as these will assemble and annotate increasingly large and complex genomes to assess greater biodiversity.

The majority of genome annotations are semi-automated, derived from informatic approaches that involve a combination of sequence alignments and *ab initio* predictions [6], [7], [8]. The inputs may include pre-assembled transcripts, raw RNA-seq reads, and closely related proteins. The resources considered depend on the available evidence, as well as the complexity and size of the genomes under investigation. The downstream genome annotations and upstream alignment files are represented in one of the more variable bioinformatic standard file formats, known as the Generic Feature Format (GFF) [9]. The GFF file provides structure for information-rich annotations as compared to the reduced representation available through the General Transfer Format (GTF). Generation of a final gene annotation requires filtering of incomplete or unlikely structural models and consideration of functional annotations at the full protein or protein domain level. The informatic packages that distill several sources of evidence into gene annotations, frequently deliver these without tools to assess their validity.

The Gene Filtering, Analysis, and Conversion (gFACs) represents a flexible annotation refining application that can accept standard annotations from primary gene annotation software as well as transcript/protein sequence aligners. In combination with the reference genome, gFACs can filter erroneous gene models, generate statistics/distributions, and provide outputs for standard downstream processing and/or visualization. Notably, this application does not replace the *ab initio* or similarity-based prediction models, but serves as a companion tool to resolve conflicting annotations and improve the quality of the final models. gFACs is unique in its ability to provide statistics and analysis, along with a direct connection to functional annotations to refine models. Similar programs such as gffread and gffcompare (https://ccb.jhu.edu/software/stringtie/gffcompare.shtml) provide gene model filtering and comparison abilities but lack application for analysis such as comprehensive statistics, functional annotation inclusion, and output standardization. gFACs can be used in tandem with these tools as it recognizes gffread inputs and provides a compatible GTF output for both gffread and gffcompare. The goal of gFACs is to aid the users in understanding their data while providing customized filters and utilities to remove and analyze gene models. As novel genomes are annotated, flexible customization and tools for analysis are essential for the tuning of final models.

## Method

**Figure 1:**
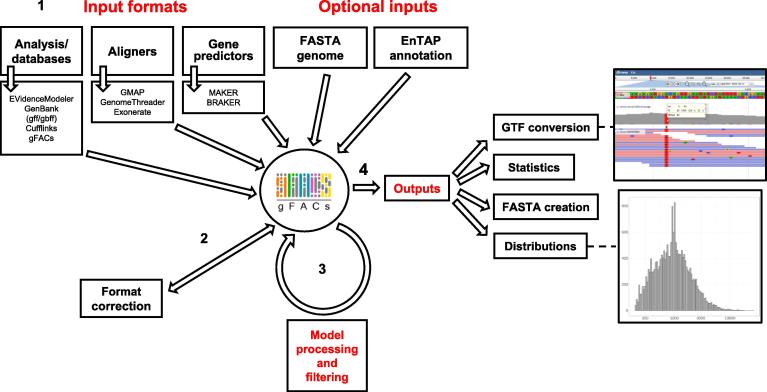
**gFACs pipeline** gFACs accepts and standardizes multiple input formats and classes with options to include functional annotation or a reference genome. Following standardization, user-specified filters modify gene models and produce various outputs for downstream analysis. Outputs may be further formatted for direct use in other software.

Accepted inputs span a range of aligners and gene predictors, which are presented in formats with similarities to GTF and GFF files. Current accepted input formats include MAKER [6], Prokka [7], BRAKER/AUGUSTUS [8], EVidenceModeler [10], GMAP [11], GenomeThreader [12], gffread, Exonerate [13], and NCBI GFF annotations. The user specifies the file source at runtime, which can be selected from an applicable set of flags. gFACs can optionally accept the reference genome in FASTA format (standard or softmasked) to permit more refined filtering and analysis. The second optional file is the annotation flat file resulting from EnTAP [14], which provides a functional annotation summary, including similarity search, protein domain, and gene family assignments for the proposed gene models or aligned sequences (Figure 1). The physical positions represented in these files are formatted into an intermediate text file to aid in processing and calculating the proposed gene space.

gFACs removes erroneous models through a set of 14 user-selected filtering options (Table 1 and Table S1), optionally aided by a reference genome or functional annotation. A notable feature of gFACs is its ability to discern and separate isoforms and conflicting gene models. This is performed by identifying overlapping exons due to conflicting evidence such as parent RNA. Splice site recognition is assumed from the provided annotation or alignment. Furthermore, gFACs is capable of collapsing these discerned isoforms and removing duplicate models to provide a final unique set. Each proposed model is subject to a predetermined set of filters as flagged by the user, many of which can be customized, such as setting minimum intron lengths or detection of in-frame stop codons. The addition of functional annotations allows for the exclusion of models without a sequence similarity search result or gene family assignment. gFACs does support alternate start codon usage, which may further increase the number of passing gene models. Alternate start sites are more common in prokaryotes (up to 20% GTG or TTG) [15] than in eukaryotes [16]. Therefore, inclusion for alternate start sites is user-specified.

**Table 1:** **Summary of gFACs filters and utilities (simplified)**

**Filter/tool**	**Description**
**Requiring GFF/GFF3/GTF input file only**	
Statistics	Generating 40 points of information on the annotation

Transcript processing	Separating alternate transcripts for independent filtering and collapsing models to unique transcripts later

Removal of incomplete genes	Identifying incomplete transcripts by missing starting or ending exons, when sequence information is unavailable

Removal of monoexonic or multiexonic genes	Separating the annotation into a monoexonic or multiexonic set through intron presence

Minimum CDS/exon/intron size	Filtering according to a default or user-specified minimum length in nucleotides

Unique transcripts	Collapsing transcripts of the same gene to the longest model and removing duplicate models

Distributions	Producing a variety of distributions and raw data creation on model data

**Requiring a FASTA file**	
Removal of genes without a start/stop codon	Filtering for a start or stop codon within each transcript and supporting alternate starts at user’s request

Removal of genes with in-frame stop codons	Filtering genes with any user-specified number of in-frame stop codons

Canonical splice sites only	Removing genes that lack the GT/AG splice type

Splice table	Reporting splice site usage

Nucleotide content	Reporting the GC/AT/N content of gene CDS

FASTA creation	Converting annotation to a protein and nucleotide FASTA file either with or without intronic sequence

Conversion and compatibility	Generating a standard GTF and providing additional arguments format to GFF for use in other software such as SnpEff and JBrowse

**Requiring an EnTAP annotation**	
Keeping genes with a similarity search or EggNOG hit	Filtering genes that lack an associated EnTAP annotation

gFACs provides a multitude of output options in addition to a log detailing the process and filtering impacts. The primary outputs include gene/protein FASTA files, GTF-represented models, comprehensive statistics on the selected gene models, and distribution tables. The distributions resulting from these filters can be easily imported in packages such as R to view gene lengths, CDS lengths, exon lengths, and exon size by order (Figure S1). Additionally, gFACs can generate annotation files that are compatible with SnpEff [17] for annotation of variants called against the genome and JBrowse for immediate import and visualization in a web-based genome browser [18].

## Implementation

Examining protein coding gene model annotations provides insight on some of the common issues associated with annotating genomes. These can include completeness (lack of start/stop or in-frame stops), gene structure (splice sites, intron/exon lengths, and mono-exonic to multi-exonic model ratios), fragmentation (incorrect start site assignment), and lack of functional validation (similarity searches, protein domains, and gene family assignments).

To demonstrate its utility, gFACs was applied to two draft public genomes for *Bos taurus* (GenBank: GCF_000003055.6) [19] and *Malus domestica* (GenBank: GCF_000148765.1) [20] with the published annotations. BRAKER v2.1.0 annotations were generated for the model *Homo sapiens* and the non-model moss, *Funaria hygrometrica*. The human BRAKER predictions were based on RNA-seq data from two libraries (Illumina 76 bp PE, UCSC: wgEncodeEH000146). Similarly*, F. hygrometrica* RNA-seq data from six libraries (Illumina 150 PE, BioProject: PRJNA421369) was provided to generate the gene annotations. Microbial application was demonstrated with a Prokka v.1.11 annotation of the *Borrelia burgdorferi* B31, (GenBank: GCF_000008685.2) [21]. Prokka predictions are derived from a basic run on only the genome, in which candidate genes are predicted by five separate tools to generate gene coordinates. All gene models (public or generated through BRAKER/Prokka) were functionally annotated with EnTAP v0.8.0, utilizing the NCBI RefSeq database and EggNOG gene family database. The models generated for *H. sapiens* and *B. burgdorferi* were evaluated against the public annotation following gFACs assessments.

A total of nine of the possible 14 filters were applied to the genomes representing unique sources: microbial, plant, and animal. These filters include removal of all genes with an intron or exon less than 20 nucleotides, CDS size minimum of 150 nucleotides, required presence of an ATG-only start and stop codon, no in-frame stop codons, canonical (GT/AG) only splice sites in multiexonic genes, and an EnTAP similarity search or gene family assignment. For *B. burgdorferi*, the canonical splice filter is not used since introns are not present. It should be noted that these filters demonstrate common issues but it would be expected that a small number of genes would have alternative splicing, micro-exons/introns, and other less common structures. These filters serve to capture clearly problematic features in these categories resulting from erroneous models.

**Figure 2:**
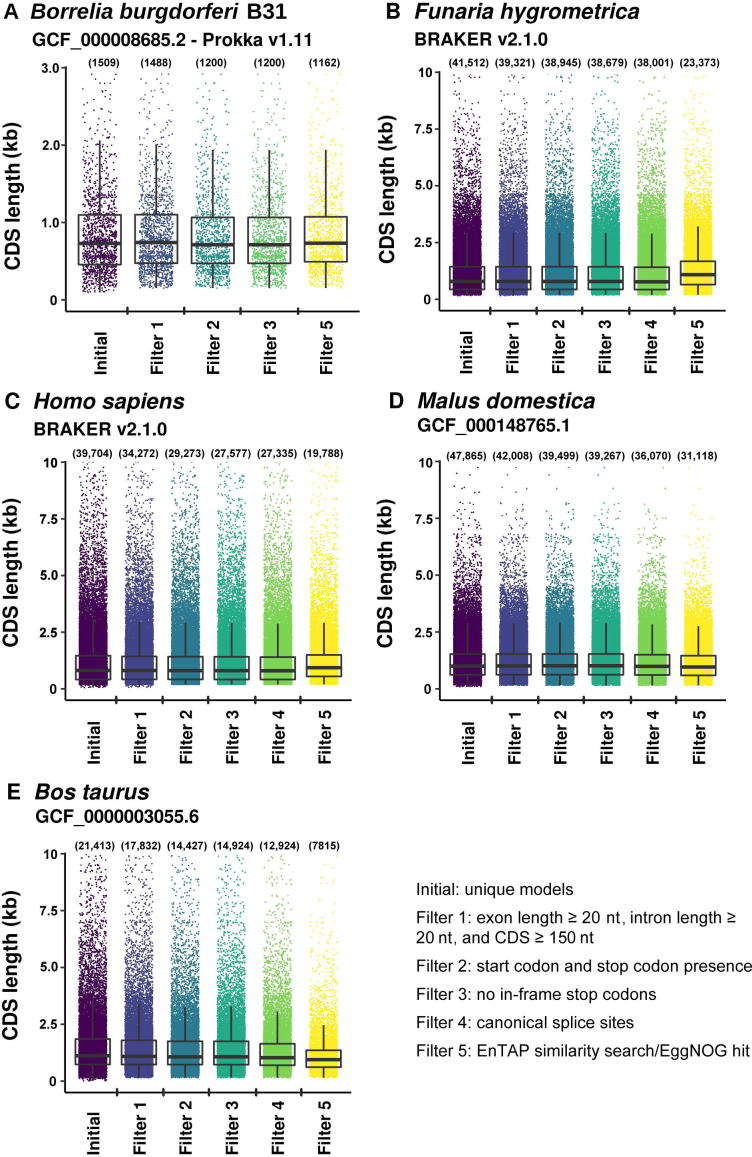
**gFACs filtration on the annotations across five species reduces the number of gene models** Nine applied filters (shown as five collective filters) reduced the number of unique genes with varied intensity. Each point represents a unique gene with quartile information demonstrated by overlapping boxplots. For viewing ease, the maximal CDS length shown on Y-axes was set as 3 kb for *B. burgdorferi* and 10 kb *for F. hygrometrica*, *H. sapiens*, *M. domestica*, and *B. taurus* (consequently, there are 85, 168, 425, 41, and 262 genes not included in the plots for *B. burgdorferi*, *F. hygrometrica*, *H. sapiens*, *M. domestica*, and *B. taurus*, respectively). The corresponding number of initial genes and the unique genes retained after filtering is provided in parenthesis on top of each column.

Runtime and memory requirements vary based on the filters applied, total initial gene models, and genome size. Run specifications among the species and filters described here (Figure 2) is recorded in (Table 2).

**Table 2:** **Summary of filtering and software requirements**

**Species**	**Genome version**	**Genome size (Mb)**	**Initial gene counts**	**Runtime**	**Memory (GB)**
*B. burgdorferi* B31	ASM868v2	1.3	1509	7 s	1
*M. domestica*	MalDomGD1.0	704	47,865	9 min 51 s	5
*H. sapiens*	hg19	3102	34,272	13 min 46 s	5

*Note*: All samples were run on 1 CPU (2.1 GHz, AMD Opteron Processor 6172). *F. hygrometrica* and *B. taurus* are not shown but perform within these ranges.

Across all species and annotations, gFACs was able to identify gene models that were potentially problematic (Figure 2). In *H. sapiens*, there were 34,272 uniquely predicted genes from BRAKER, which was reduced to 19,768 using the applied filters (27,336, without considering functional annotation filter). The number of uniquely predicted genes after filtering (19,768) is comparable to the number of protein-coding genes (20,203) of the latest human genome annotation (GRCh38p.12). Detailed analysis reveals there is a larger proportion of predicted genes that match to the reference gene in the unfiltered annotation (22.4%) compared to the 19,768 genes with filtered annotation (12.5%). In terms of the match types, the proportion of perfect matches increased from 17.0% (unfiltered) to 22.1% (filtered). The proportion of perfect and contained matches increased from 26.3% (unfiltered) to 33.8% (filtered). Finally, the proportion of all types of matches increased, from 65.5% (unfiltered) to 69.3% (filtered). To further demonstrate the abilities of gFACs, a more comprehensive filtering on *H. sapiens* was performed (Table S1). These additional steps show the reduction in gene models in the software’s default order. Additional annotation statistics, such as splice usage, nucleotide content, and 41 statistics quantifying the final annotation are also included.

Similarly, *B. burgdorferi* gene predictions were reduced from 1509 to 1162 models, which represents an improvement compared to the public annotation of 1208 models (94.4% perfect matches and 98.9% total matches). Inclusion of alternative start codons in *B. burgdorferi* increases the number of passing models to 1416 (93.2% perfect matches and 98.1% total matches), which may include a small number of erroneous models.

The *F. hygrometrica*, *B. taurus*, and *M. domestica* models show similar rates of reductions through all filters including functional annotation at 43.70%, 34.99%, and 63.50%, respectively (Figure 2). The extent of model match in these species is not assessed due to the lack of a rigorous annotation. However, we noted an improvement of the models in terms of removing biologically unlikely exon, intron, and CDS lengths.

## Conclusion

In summary, the gFACs software package provides a comprehensive framework for evaluating, filtering, and analyzing gene models from a range of input applications and preparing these annotations for formal publication or downstream analysis. We hope to meet the needs of the growing enthusiasm to annotate new species with a software that provides greater utility to a complex process.

## Availability

gFACs is freely available and implemented in Perl with support from BioPerl libraries at https://gitlab.com/PlantGenomicsLab/gFACs.

## Authors’ contributions

MC developed the software package and performed all analysis in the manuscript. MC drafted and revised with help from JW. Both authors read and approved the final manuscript.

## Competing interests

The authors have declared no competing interests.

## Figures and Tables

**Supplementary figure 1:**