# Supplementary material for: gFACs: Gene Filtering, Analysis, and Conversion to Unify Genome Annotations Across Alignment and Gene Prediction Frameworks
Source: Genomics Proteomics Bioinformatics. 2019 Aug 19;17(3):305–10. doi: 10.1016/j.gpb.2019.04.002 (PMC6818179; doi:10.1016/j.gpb.2019.04.002)
Supplement: Supplementary data 1 [file mmc1.docx]

**Table S1 Extensive gFACs filtering and statistics on the BRAKER 2.1.0 annotation of *Homo sapiens***

| Filter / Analysis steps | | Results | | |
| --- | --- | --- | --- | --- |
| No. of original input protein-coding genes | | 34,273 | | |
| No. of genes with overlap (splice/conflicting models) | | 4051 | | |
| No. of transcripts created from overlap | | 9482 | | |
| No. of models (overlap + non-overlap) | | 39,704 | | |
| Filter: Models that are complete at 5' end | | 38,101 | | |
| Filter: Models that are complete at 3' end | | 36,579 | | |
| Filter: Models that do not have an exon < 20 nt | | 30,937 | | |
| Filter: Models that do not have an intron < 20 nt | | 30,937 | | |
| Filter: Models that do not have a CDS < 150 nt | | 30,937 | | |
| Filter: Models that have an EnTAP similarity search/ EggNOG hit | | 19,768 | | |
| Filter: Models that have only canonical splice sites (if multiexonic) | | 19,768 | | |
| Filter: Models that have an ATG start codon | | 19,768 | | |
| Filter: Models that have an ending in-frame stop codon | | 19,768 | | |
| Filter: Models that have no additional in-frame stop codons | | 19,768 | | |
| Filter: Unique models (collapsed isoforms) | | 19,768 | | |
| Analysis (gathered from an identical run without the canonical only filter) | Splice site type | GC/AG | AT/AC | GT/AG |
|  | Usage count | 199 | 64 | 149,957 |
|  | Overall use percentage | 0.13% | 0.04% | 99.83% |
| Analysis: Nucleotide content of CDS | GC content | 52.44% | | |
|  | AT content | 47.56% | | |
|  | N content | 0% | | |
| Statistics | |  | | |
| No. of genes | | 19,768 | | |
| No. of monoexonic genes | | 5761 | | |
| No. of multiexonic genes | | 14,007 | | |
| No. of positive strand genes | | 10,675 | | |
| No. of positive strand monoexonic genes | | 3394 | | |
| No. of positive strand multiexonic genes | | 7281 | | |
| No. of negative strand genes | | 9093 | | |
| No. of negative strand monoexonic genes | | 2367 | | |
| No. of negative strand multiexonic genes | | 6726 | | |
| Average overall gene size (nt) | | 28,447.56 | | |
| Median overall gene size (nt) | | 8640 | | |
| Average overall CDS size (nt) | | 1241.41 | | |
| Median overall CDS size (nt) | | 933 | | |
| Average overall exon size (nt) | | 200.12 | | |
| Median overall exon size (nt) | | 129 | | |
| Average size of monoexonic genes (nt) | | 766.30 | | |
| Median size of monoexonic genes (nt) | | 660 | | |
| Largest monoexonic gene (nt) | | 11,862 | | |
| Smallest monoexonic gene (nt) | | 201 | | |
| Average size of multiexonic genes (nt) | | 39,828.59 | | |
| Median size of multiexonic genes (nt) | | 19,494 | | |
| Largest multiexonic gene (nt) | | 1,031,336 | | |
| Smallest multiexonic gene (nt) | | 303 | | |
| Average size of multiexonic CDS (nt) | | 1432.71 | | |
| Median size of multiexonic CDS (nt) | | 1080 | | |
| Largest multiexonic CDS (nt) | | 26,013 | | |
| Smallest multiexonic CDS (nt) | | 201 | | |
| Average size of multiexonic exons (nt) | | 171.72 | | |
| Median size of multiexonic exons (nt) | | 125 | | |
| Average size of multiexonic introns (nt) | | 5228.52 | | |
| Median size of multiexonic introns (nt) | | 1527 | | |
| Average No. of exons per multiexonic gene | | 8.34 | | |
| Median No. of exons per multiexonic gene | | 6 | | |
| Largest exon in a multiexonic gene (nt) | | 14,719 | | |
| Smallest exon in a multiexonic gene(nt) | | 20 | | |
| Highest No. of exons in one gene | | 148 | | |
| Average No. of introns per multiexonic gene | | 7344 | | |
| Median No. of introns per multiexonic gene | | 5 | | |
| Size of largest intron (nt) | | 317,318 | | |
| Size of smallest intron (nt) | | 48 | | |

*Note:* Filter and analysis steps are presented in the default order performed. The step of unique transcript filter (isoform collapse) is performed at last by default whereas it is performed at first in implementation (Figure 2). This may slightly alter the final numbers. All size statistics are in nucleotide length (nt).
